# Estimation of Serum Ferritin in Mucormycosis Patients and Prognostication Based on the Ferritin Value

**DOI:** 10.7759/cureus.24013

**Published:** 2022-04-10

**Authors:** Babu Anand C, Sivasubramaniyam Senthilkumar, Nivedha P, Mohammed Ibrahim C, Khizer Hussain Afroze M, Ramanathan M

**Affiliations:** 1 General Medicine, K.A.P. Viswanatham Government Medical College, Tiruchirappalli, IND; 2 Anatomy, MVJ (M.V. Jayaraman) Medical College and Research Hospital, Bangalore, IND

**Keywords:** diabetes mellitus, ferritin, antifungal medications, fungal infection, covid-19, mucormycosis

## Abstract

Introduction: Coronavirus disease 2019 (COVID-19) caused by the severe acute respiratory syndrome coronavirus 2 (SARS-CoV-2) has been implicated in a variety of vulnerable bacterial and fungal diseases. Mucormycosis is a life-threatening infection caused by fungi belonging to the class Zygomycetes and the order Mucorales. The aim of the present study is to evaluate the level of serum ferritin level in mucormycosis patients and to prognosticate them based on those values.

Materials and methods: This prospective observational study was conducted in the Department of General Medicine, Mahatma Gandhi Memorial Government Hospital, Tiruchirappalli, in 50 diagnosed mucormycosis patients.

Results: During the study period, 44 had prior COVID-19 illness (post-COVID). Six patients had mucormycosis with no prior COVID-19 illness. Rhino-orbital involvement was found in 44 of the 50 cases, with three of them having cerebral extension. Forty-one cases recovered and were discharged, six cases remained sick and were hospitalized, and three died. The post-COVID patients (554.13 ± 371.60) have greater serum ferritin levels than non-COVID patients (259.95 ± 110.15), which are statistically significant.

Conclusion: Mucormycosis patients tend to have higher serum ferritin values, especially among non-survivors and sick patients than survivors. For a better chance of recovery and survival, early identification, surgical debridement, and antifungal medications are essential.

## Introduction

Mucormycosis is a life-threatening infection caused by fungi belonging to the class Zygomycetes and the order Mucorales. The most prevalent cause of infection is fungi from the Mucoraceae family, notably the species *Rhizopus oryzae*. Iron availability has been indicated as a major regulator of Zygomycetes' pathogenicity based on strong clinical data [1,2].

Coronavirus disease 2019 (COVID-19) caused by the severe acute respiratory syndrome coronavirus 2 (SARS-CoV-2) has been implicated in a variety of vulnerable bacterial and fungal diseases [1]. Fever, cough, dyspnea, and other nonspecific prodromal symptoms like myalgia and anosmia were common in the COVID-19 patients, who may progress to severe respiratory distress syndrome. The incidence of ocular and cerebral problems attributable to fungal etiology was not found in the first wave of COVID-19 [3].

In the second wave (in India between February 2021 and July 2021), both active and recovered COVID patients had rhino-orbital cerebral mucormycosis [4]. Several theories have been postulated to explain this sudden increase in mucormycosis cases: 1) COVID-19-related immunological dysregulation and aberrant iron metabolism; 2) COVID-19-related immune dysfunction and hyperglycemia due to steroid therapy; 3) concurrent uncontrolled diabetes [3].

Hence, the present study is designed to evaluate the level of serum ferritin level in mucormycosis patients and prognosticate them based on the ferritin values, and also to evaluate the mucormycosis patients based on COVID and non-COVID status.

## Materials and methods

Study design

After acquiring the approval from the institutional ethics committee of KAPV Government Medical College with Ref. No. KAPV/IEC/2021/032, this prospective observational study was conducted for a period of three months from June 2021 to September 2021. This research was performed in the Department of General Medicine, Mahatma Gandhi Memorial Government Hospital, Tiruchirappalli (Trichy).

Study population

All the inpatients who were diagnosed with mucormycosis through tissue biopsy were enrolled and followed for 12 weeks.

Inclusion criteria

1. Patients greater than 18 years of age.

2. Tissue biopsy-proven cases of mucormycosis.

3. Patients/attendants who have given written informed consent.

4. Reverse transcriptase-polymerase chain reaction (RT-PCR) negative for COVID-19 at the time of inclusion.

Exclusion criteria

1. Patients who had chronic immunosuppressant therapy.

2. Patients with a history of recent iron formulation therapy.

3. Patients who had recently received voriconazole therapy (which is used to treat aspergillosis, which is a close mimicker to mucormycosis; however, voriconazole usage can aggravate the mucormycosis worse).

4. Patients with a history of hematological malignancies on follow-up.

5. Post-organ-transplant patients on follow-up.

6. Active COVID-19 cases since they are associated with elevated inflammatory markers but we have included the post-COVID cases in our study.

Treatment with iron formulations was not included as iron has been associated with more incidences of mucormycosis infections. Patients with hematological malignancies and post-organ-transplant patients were also excluded as they were treated with chronic immunosuppressant drugs.

Methods

All inpatients diagnosed with mucormycosis by tissue biopsy were selected and followed for three months. Depending on the stage/involvement, they underwent functional endoscopic sinus surgery, maxillectomy, or orbital exenteration during their hospital stay. All the patients were treated with an injection of liposomal amphotericin B 5 mg/kg (8 mg-10 mg/kg for cerebral extension) intravenous once daily for two weeks and followed by oral tablets. Posaconazole 100 mg TDS for eight weeks or more as per clinical recovery. During the follow-up period, they were reviewed regularly by the ENT, Ophthalmology, Dental, and General Medicine specialists for improvement or disease progression. All the patients were subjected to fasting blood glucose, 2 hours postprandial blood glucose, and complete blood count. Serum ferritin and C-reactive protein (CRP) were taken during the time of admission and those values were considered for analysis. Meanwhile, improvement in symptoms, recurrence, and death was noted. Those who encountered a recurrence of symptoms were considered sick, whereas those who were discharged without symptoms were considered recovered. Serum ferritin was tested by the chemiluminescent immunoassay method (the normal range for males was 22-322 ng/mL; for females 10-291 ng/mL). CRP was done by the quantitative turbidometry method (normal value <10 mg/L).

Case definitions

**Figure 1:**
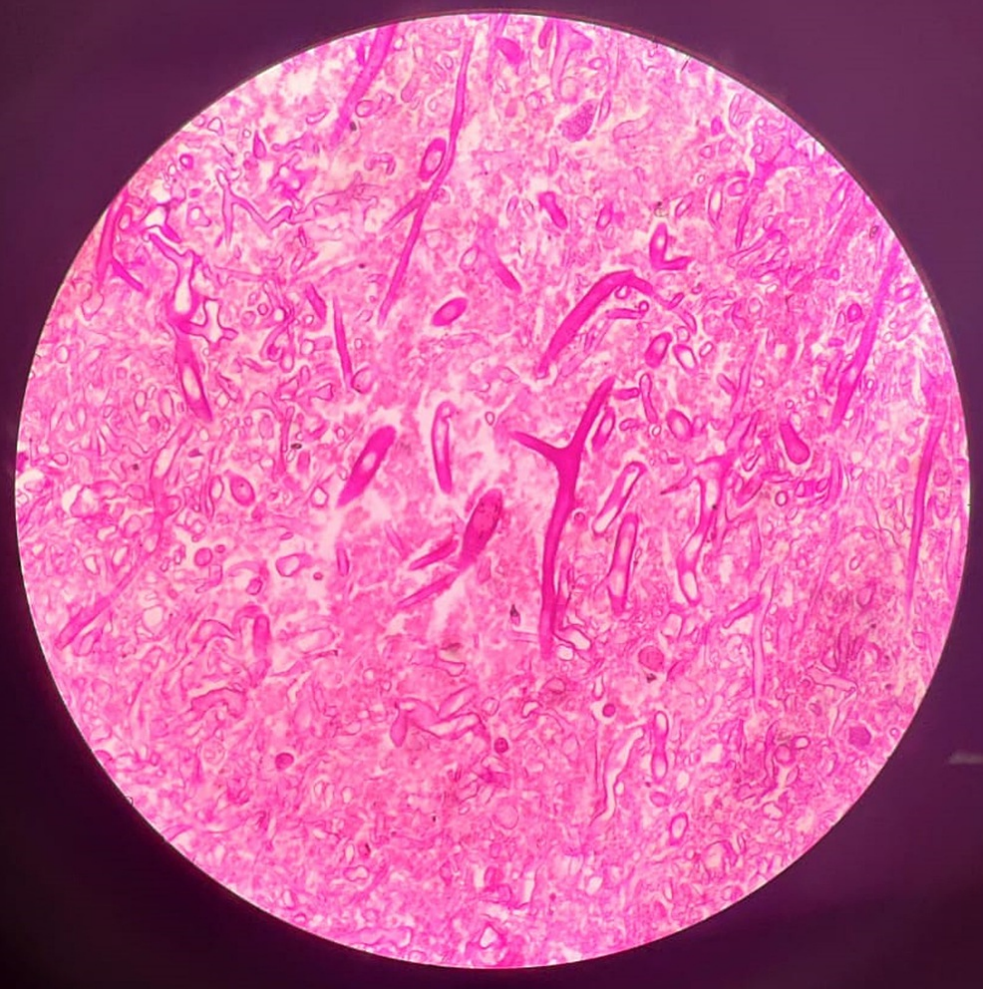
Tissue biopsy picture showing branched aseptate hyphae suggestive of mucormycosis

Cases - tissue-biopsy-proven mucormycosis patients; diabetes - based on the previous history and fasting and postprandial blood sugars; prognostic outcome - based on recovery, sickness, and death; recovery (survivor patients) means a complete resolution of symptoms with no recurrence in three months; sick - those patients who are still symptomatic, hospitalized, and those who have recurrences. Death (non-survivor) means patients who died of mucormycosis-related complications. Figure 1 shows a tissue biopsy image with branched aseptate hyphae, suggestive of mucormycosis.

Statistical analysis

The data obtained were analyzed by using SPSS for Windows, version 16.0 (SPSS Inc., Chicago, IL, USA). The Chi-square test was applied to check for an association between categorical variables. Analysis of variance (ANOVA) has been used to find the significance of study parameters between three or more independent groups.

## Results

Demography

During the study period, 44 (88%) patients had prior COVID-19 (based on past medical records). Six (12%) patients with non-COVID (based on RT-PCR negative) mucormycosis were included. Thirty-five patients out of 44 in the post-COVID group were exposed to steroids in the form of injection dexamethasone 8 mg IV OD for 7-14 days. The mean age of the study population was 53.18 ± 9.13 years, among post-COVID patients, it was 52.77 ± 9.31 years, and among non-COVID patients, it was 56.17 ± 7.68 years (p-value = 0.398). The age group of 46-50 years had the most cases (58%), followed by the age groups of 31-45 years and >60 years (22%).

Comorbidities

Thirty-three patients were known diabetic and were hypertensive and had renal failure. Other 17 patients were incidentally diagnosed to have elevated serum glucose levels during the hospitalization. 

Clinical features

**Figure 2:**
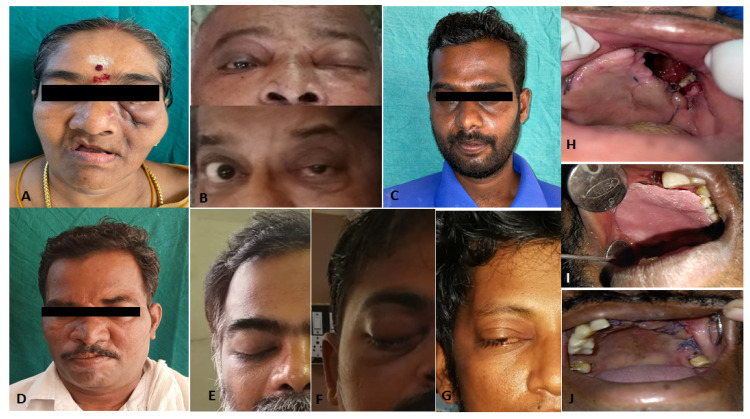
Some of the images of the patients (A-J) before and after the treatment

Forty-six of the 50 patients had rhino-orbital involvement, out of which three had a cerebral extension. Forty-one cases recovered and were discharged, six cases remained sick and were hospitalized till the end of the study period, and three died. Figure 2 shows some of the images of the patients (A-J) before and after the treatment. 

Laboratory values

The mean ferritin value of the study population was 518.83 ng/mL, among post-COVID patients it was 554.13 ± 371.60, and among non-COVID patients, it was 259.95 ± 110.15 (p-value = 0.049). Serum ferritin tends to be higher in the post-COVID group. 

Comparison between COVID and non-COVID group

Characteristics of the study population and comparison of COVID and non-COVID groups, survivor, non-survivor, and sick patients are tabulated in Tables 1, 2, respectively.

**Table 1:** Characteristics in the study population and the COVID and non-COVID patients. COVID, coronavirus disease; WBC, white blood cells.

Parameters		Post-COVID patients (n = 44)	Non-COVID patients (n = 6)	Overall (n = 50)	p-Value
Age (years)		52.77 ± 9.31	56.17 ± 7.68	53.18 ± 9.13	0.398
Sex	Male	31 (70.5%)	2 (33.3%)	33 (66%)	0.72
	Female	13 (29.5%)	4 (66.7%)	17 (34%)	
Duration of stay (in days)		22.33 ± 9.31	24.27 ± 10.10	24.04 ± 9.94	0.659
Laboratory investigations (mean ± SD)					
WBC (counts/µL)		14500 ± 3798.95	12995.68 ± 6009.79	13176.19 ± 5780.27	0.555
Polymorphonuclear leukocytes (%)		75.83 ± 8.93	75.25 ± 11.175	75.32 ± 10.852	0.903
Lymphocytes (%)		16.50 ± 8.17	17.20 ± 10.238	17.12 ± 9.942	0.873
Ferritin (ng/mL)		554.13 ± 371.60	259.95 ± 110.15	518.83 ± 362.96	0.049
Average glucose (mg/dL)		395.77 ± 66.02	307.81 ± 64.78	318.36 ± 70.43	0.003
Neutrophil-to-lymphocyte ratio		6.02 ± 3.76	7.06 ± 5.97	6.93 ± 5.73	0.681
Platelets (lakhs/µL)		4.09 ± 1.09	3.76 ± 1.43	3.8 ± 1.39	0.597
Hemoglobin (g/dL)		11.2 ± 2.44	11.33 ± 1.85	11.31 ± 1.9	0.877
Urea (mg/dL)		46.33 ± 23.347	27.59 ± 8.11	29.84 ± 12.30	0.000
Creatinine (mg/dL)		1.57 ± 1.30	0.88 ± 0.22	0.96 ± 0.51	0.001
Site of mucormycosis					
Rhino-orbital					
Yes		41 (82%)	5 (10%)	46 (92%)	0.404
No		3 (6%)	1 (2%)	4 (8%)	
With cerebral Involvement		3 (6%)	-	3 (6%)	

**Table 2:** Characteristics of the study population and among survivor, non-survivor, and sick patients. COVID, coronavirus disease; WBC, white blood cells; CRP, C-reactive protein.

Parameters	Survivor/recovered patients (n = 41)	Non-survivor/death (n = 3)	Sick (n = 6)	p-Value
Post-COVID				
No	5 (83.3%)	1 (16.7%)	0 (0%)	0.348
Yes	36 (81.8%)	2 (4.5%)	6 (13.6%)	
CRP (mg/L)				
<150	39 (95.1%)	1 (2.4%)	1 (2.4%)	0.000
150-199	1 (33.3%)	0 (0%)	2 (66.7%)	
>200	1 (16.7%)	2 (33.3%)	3 (50%)	
Neutrophil-to-lymphocyte ratio (mean ± SD)	6.21 ± 4.64	8.73 ± 8.10	10.94 ± 9.89	0.144
<3	14 (87.5%)	1 (6.2%)	1 (6.2%)	0.715
3-9	16 (80%)	1 (5%)	3 (15%)	
9-18	10 (83.3%)	1 (8.3%)	1 (8.3%)	
>18	1 (50%)	0 (0%)	1 (50%)	
Ferritin (ng/mL) (mean ± SD)	419.29 ± 250.97	772.5 ± 437.43	1072.13 ± 472.70	0.000
<300	11 (91.7%)	0 (0%)	1 (8.3%)	0.001
301-500	18 (94.7%)	1 (5.3%)	0 (0%)	
501-1000	11 (84.6%)	1 (7.7%)	1 (7.7%)	
>1000	1 (16.7)	1 (16.7%)	4 (66.7%)	
Duration of stay (in days)				
8-14 days	11 (91.7%)	1 (8.3%)	0 (0%)	0.150
15-21 days	13 (81.2%)	2 (12.5%)	1 (8.2%)	
>21 days	17 (77.3%)	0 (0%)	5 (22.7%)	
WBC (counts/µL) (mean ± SD)	13136.59 ± 5054.44	16866.67 ± 3808.32	11601.67 ± 10300.14	0.443
<11000	20 (87.0%)	0 (0%)	3 (13.0%)	0.256
>11000	21 (77.8%)	3 (11.1%)	3 (11.1%)	

## Discussion

In India, diabetes is the most common comorbidity associated with mucormycosis, accounting for 73.5% of cases [5]. However, hyperglycemia is linked to 17% of mucormycosis cases in Western countries [6]. Mucormycosis is found in about 1.6 instances per 1000 diabetic people [7,8]. In our study, diabetes was found in all the patients with mucormycosis. Thirty-three patients were already diabetic and 17 were newly diagnosed diabetic individuals.

Malignancy or neutropenia has been related to greater mortality rates in previous studies [6,9-11]. Patients with higher baseline iron or ferritin concentrations had a higher chance of mortality. As a result, the relationship between iron and ferritin concentrations and mortality may indicate increased baseline iron storage in patients, leading to more severe infection, underlying illness, or both [11-15].

Similar to this study, Brad Spellberg et al. found that neutropenia and high ferritin levels upon admission were associated with increased mortality. Deferasirox-AmBisome therapy for mucormycosis (DEFEAT Mucor) was the first randomized clinical trial in mucormycosis patients, which showed that adjunctive deferasirox did not improve outcomes in mucormycosis. It was based on the concept that increased iron overload was associated with increased growth of mucormycosis [11].

Neutropenia is the risk factor for mucormycosis but we see neutrophilia in COVID-19, and lymphopenia seen in COVID-19 does not seem to be a risk factor because of the low incidence of mucormycosis among the lymphopenic syndromes and HIV patients [16]. The neutrophil-to-lymphocyte ratio (NLR) has been linked to a higher incidence of acute renal damage and mortality, according to Guney BC et al. [17-20]. Gameiro et al. found a link between the NLR and death in septic renal failure patients in similar research [18].

The general concept of iron is that it exists in two ionization states: Fe^2+^ (ferrous) and Fe^3+^ (ferric). Iron can donate and accept electrons due to its ability to exist in either of these two forms and hence can participate in a wide range of cellular oxidation-reduction reactions. Iron acquisition is the critical step in the pathogenesis of mucormycosis that can happen by three mechanisms: i) by reduction from ferric to ferrous form and subsequent transport by permease [21-24]; ii) through siderophore-based pathway [25-27]; iii) through hemin [28]. Deferoxamine acting as a siderophore for iron acquisition contributed to the increased prevalence of mucormycosis in renal failure patients receiving deferoxamine medication [3].

Iron is potentially toxic as such because of its ability to produce free radicals through the Fenton or Haber-Weiss reaction. So it is stored in proper storage form to prevent its toxicity. Iron can be stored as ferritin, which is a protein-bounded form. Zygomycetes can store iron as ferritin [3].

When comparing non-survivors with survivors, Yan et al. discovered a greater ferritin level in severe COVID-19 patients [29]. According to a meta-analysis conducted by Cheng L et al., ferritin level fluctuations in COVID-19 patients are not only higher in non-survivors than in survivors, but they also increase as the disease progresses. They also revealed that non-survivors with COVID-19 had considerably greater ferritin levels than survivors with COVID-19 [30]. Serum ferritin of more than 1000 ng/mL was associated with increased morbidity in terms of hospital stay and recovery time as shown by Bhanuprasad K et al., and serum ferritin was significantly greater in mucormycosis patients and was also significantly connected to mortality [31]. Another study conducted by Bhadania S et al. recorded a significant difference in serum ferritin levels between mild (193.6 ng/mL) and severe invasive mucormycosis (342.1 ng/mL) [32]. 

We also found that mean serum ferritin was found to be higher in post-COVID-associated mucormycosis (554.13 ± 371.60 ng/mL) than in non-COVID individuals with mucormycosis (259.95 ± 110.15 ng/mL) in this investigation, which was statistically significant.

During COVID-19 pneumonia, increased ferritin is released into the blood as an inflammatory response [33]. Ferritin is the storage form of iron, which protects the cell from its toxic effects. When ferritin is released into the circulation, it releases its inner iron leading to increased serum free iron which further triggers the liver to produce further ferritin, so it continues in a vicious cycle [34]. Also, SARS-CoV-2 spike proteins themselves mimic the hepcidin causing dysregulation of iron metabolism leading to hyperferritinemia and ferroptosis independent of inflammation [35]. 

Limitations of the study

The small sample size was a major limitation in our study. We recommend that larger sample sizes be used in future studies to validate the results of the present study. In this study, we did not compare any other iron parameters.

## Conclusions

Mucormycosis patients tend to have higher serum ferritin values, especially among non-survivors (death) than survivors (recovered). The prognosis and outcome of the mucormycosis can also be assessed based on the serum ferritin values. The surge of mucormycosis cases among the COVID-19 patients can also be explained by higher ferritin values, which is a marker of altered iron metabolism. It is a proven fact that altered iron metabolism and high iron storage are promoting factors for fungal infections like mucormycosis. It is also noted in our study that early identification of mucormycosis followed by surgical debridement and antifungals had a good outcome.
